# Adipose tissue ceramides: emerging lipid components of extracellular vesicles and their influence on metabolic dysregulation

**DOI:** 10.3389/fendo.2026.1919367

**Published:** 2026-09-09

**Authors:** Sharon Angelini, Juana Maria Sanz Molina, Fabiola Castaldo, Nenad Naumovski, Angelina Passaro, Domenico Sergi

**Affiliations:** 1 Department of Translational Medicine, University of Ferrara, Ferrara, Italy; 2 Food, Chemical and Biotechnology Cluster, Singapore Institute of Technology, Punggol, Singapore; 3 School of Medicine and Psychology, Australian National University, Canberra, ACT, Australia; 4 Department of Nutrition and Dietetics, School of Health Science and Education, Harokopio University, Athens, Greece; 5 Discipline of Nutrition and Dietetics, Faculty of Health, University of Canberra, Canberra, ACT, Australia

**Keywords:** adipose tissue, ceramides, extracellular vesicles, insulin resistance, lipid metabolism, obesity

## Abstract

Adipose tissue dysfunction is at the interphase between obesity and impaired metabolic health. Increased lipid spillover is a key mechanism by which adipose tissue dysfunction contributes to systemic metabolic dysregulation. This, in turn, leads to the accumulation of lipotoxic lipid species, such as ceramides, within metabolically active tissue not suited for lipid storage tissues thereby fueling insulin resistance. However, the buildup of ceramides also occurs in the adipose tissue of individuals with obesity where these sphingolipids may disrupt energy metabolism, insulin sensitivity and affect adipose tissue secretome, compromising systemic metabolic health. This review will provide an updated overview on the contribution of ceramides to adipose tissue dysfunction, with a particular focus on the onset of insulin resistance. Additionally, this manuscript will focus on the involvement of ceramides in modulating extracellular vesicle (EVs) secretion from the white adipose tissue and their role as bioactive components of EV lipidome able to discern individuals based on their metabolic status.

## 1 Introduction

The global health and economic burden of obesity is still impetuously on the rise. Over 1 billion individuals worldwide were living with obesity in 2021, with its prevalence being forecasted to double by the year 2050 (1). The health and socio-economic impact of obesity is the direct consequence of the cardio-metabolically detrimental effect of excess fat deposition. In particular visceral adiposity, is a pivotal risk factor for the development of potentially fatal comorbidities, including type 2 diabetes mellitus (T2DM), cardiovascular and neurodegenerative diseases as well as several types of cancer (2). Body weight gain and the consequent increase in adiposity are the result of a long-term positive energy balance, with fuel excess being stored in the form of triglycerides within adipocytes. However, while the adipose tissue is instrumental in buffering excess metabolic substrate availability, long-term energy oversupply, leading to adipocyte hypertrophy, hampers adipose tissue metabolic and endocrine function (3, 4). These pathophysiological changes underlay adipose tissue dysfunction and are marked by an impairment in adipocyte energy buffering capacity, insulin resistance and the activation of inflammatory responses. The latter is driven by adipocytes as well as adipose tissue resident and infiltrating immune cells (5). Additionally, adipose tissue dysfunction is characterized by a disruption in its endocrine, secretory function. This results in an increase in the secretion of adipokines, miRNAs and a plethora of other metabolites that are able to disrupt systemic metabolic health (6). In parallel, the derangement of adipose tissue endocrine function promotes a decline in the mediators exerting metabolically beneficial effects, which contributes to exacerbating metabolic deregulation (6). This decline in adipose tissue metabolic health is also accompanied by a shift in the size, number and cargo of the extracellular vesicles (EVs) it secretes (7, 8). EVs, in turn, are a key component of adipose tissue secretome (9). As such, EVs play a pivotal role in mediating the cross-talk between the adipose tissue and other metabolically active organs, contributing to shaping systemic metabolic health (10–12).

Although fuel excess is stored in the form of triglycerides within adipocytes, a portion of energy overload can be channeled towards ceramide synthesis. This shift in energy partitioning can be observed in individuals with obesity who display an increase in ceramide levels within the adipose tissue (13). In this regard, increased ceramide accumulation within the adipose tissue may contribute to its dysfunction by fostering insulin resistance, disrupting lipid metabolism and promoting metabolic inflammation referred to as a sterile, low-grade chronic inflammatory status elicited by metabolic stressors (14–16). Additionally, ceramides are directly involved in modulating EV secretion (17) and are also enriched in the EVs secreted from the adipose tissue of individuals with obesity and mirror the metabolic status of the individual (18). Therefore, ceramide accumulation within extra adipose metabolically active tissues, such as the liver and skeletal muscle, may not be the only discriminant mediating the dysmetabolic effects of these sphingolipids. This review aims at providing a comprehensive and updated overview of the role of ceramide buildup within adipocytes on white adipose tissue as well as systemic metabolic dysfunction. This manuscript will also give an overview about the role of white adipose tissue-derived EVs as emerging carriers of ceramides and the contribution of these sphingolipids in modulating the EV secretion.

## 2 Ceramide metabolism

**Figure 1:**
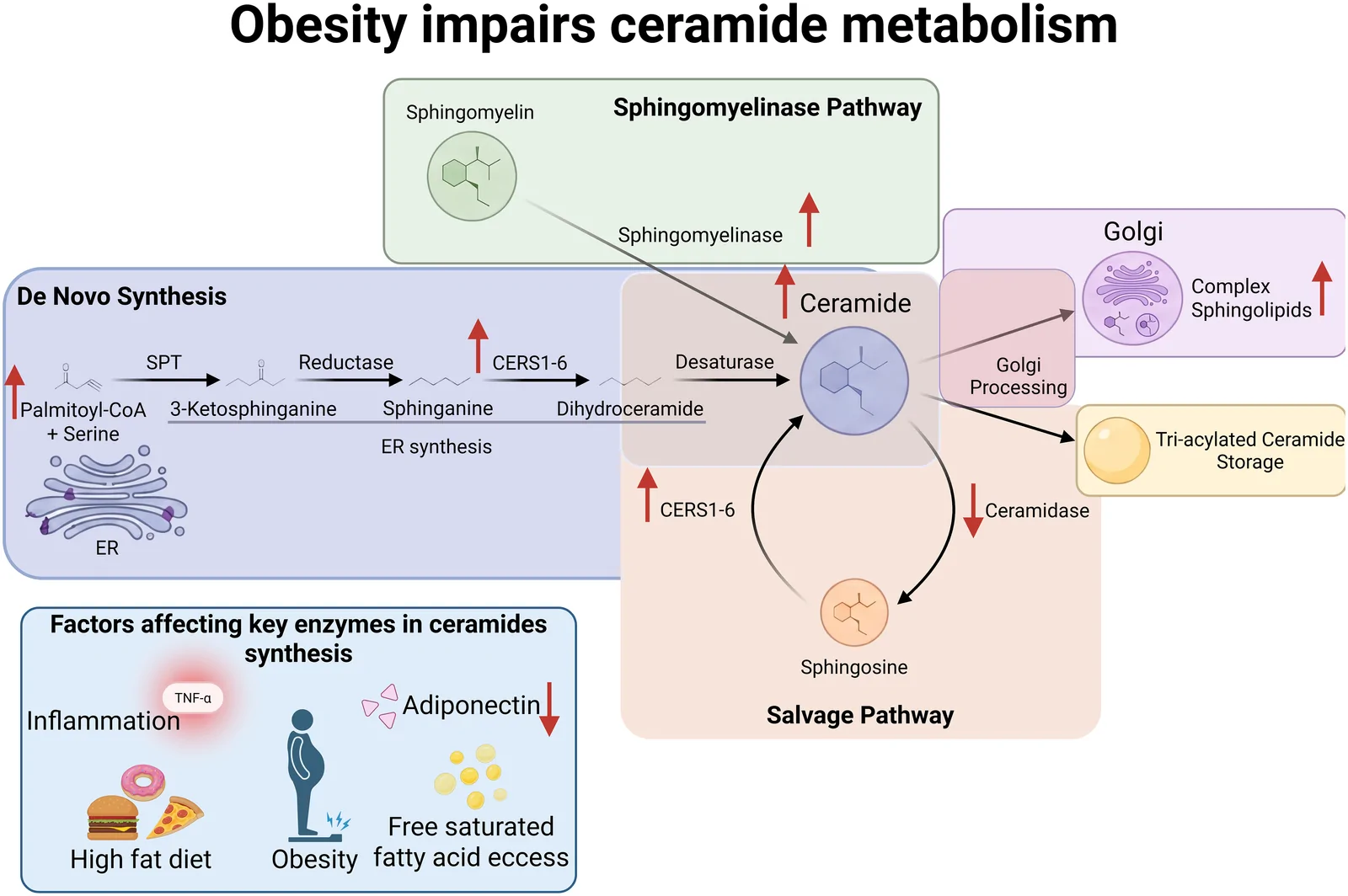
Obesity alters ceramides metabolism. Schematic representation of ceramide synthesis and keyenzymes involved in *de novo* synthesis, sphingomyelinase and salvage pathways. Redarrows indicate enzymes whose activity/expression is induced (upwards arrows), downregulated or inhibited (downwards arrows) in obesity. ER, endoplasmic reticulum; SPT, serine palmitoyl transferase; CERS1-6, ceramide synthase 1-6; TNFα, tumor necrosis factor alpha. Image created with BioRender.com.

Ceramides are sphingolipids made up of a sphingosine backbone linked to a fatty acid of different length *via* an amide bond (19). Despite their lower abundance compared to glycerolipids within the cellular lipidome, ceramides are actively involved in regulating several cellular processes including cell proliferation, apoptosis, inflammation, fuel metabolism and energy balance *via* the hypothalamus (16, 20–23). Furthermore, ceramides have a structural role being an integral component of cell membranes involved in regulating their stability and the distribution of surface receptors (24). Given the role of ceramides as bioactive lipids, alterations in their cellular homeostasis will impact upon cellular pathophysiology (16, 25). Ceramide can be synthesized *de novo*, derived from sphingomyelin catabolism or regenerated *via* the salvage pathway (**Figure 1**) (16, 26).

The *de novo* synthesis of ceramide occurs on the cytosolic surface of the endoplasmic reticulum. It initiates with the condensation of palmitoyl Coenzyme A (CoA) with the amino acid serine through a reaction catalyzed by serine palmitoyltransferase (SPT) to produce 3-ketosphinganine (**Figure 1**). Successively, 3-ketosphinganine reductase converts 3-ketospinganine to sphinganine which is N-acylated by a family of six ceramide synthases (CERS1–6). This results in the production of dihydroceramides with variable acyl chain lengths spanning form 14 to 34 carbons (27). The final step of the *de novo* ceramide synthesis is mediated by dihydroceramide desaturases which insert a 4,5-trans-double bond into the sphingoid backbone of the dihydroceramides (**Figure 1**). Once synthetized, ceramides are transferred to the Golgi apparatus where they are used as substrates for the synthesis of complex sphingolipids. Furthermore, ceramides can be further acylated with this new formed tri-acylated ceramide being stored in lipid droplets (**Figure 1**) (28).

Ceramides can also be produced *via* the catabolism of sphingomyelin and the salvage pathway. The catabolism of sphingomyelin involves its degradation by sphingomyelinases with the subsequent release of ceramide and the choline head group (29) (**Figure 1**). In contrast, the salvage pathway regenerates ceramides *via* CERS-mediated acylation of the sphingosine which, in turn, derives from the breakdown of ceramides by ceramidases (**Figure 1**) (30).

Ceramides are catabolized *via* a ceramidase-mediated diacylation. The resulting sphingosine is phosphorylated to generate spingosine-1-phosphate and then degraded in the endoplasmic reticulum by sphingosine-1-phosphate lyase (31) (**Figure 1**). Besides the five ceramidases identified in mammals and classified as acid, alkaline and neutral (27), the adiponectin receptor also harbor ceramidase activity (32).

Several factors have been shown to promote ceramide synthesis by interfering with key enzymes involved in the pathways described above. For example, high-fat feeding and inflammation has been reported to upregulate enzymes involved in the *de novo* ceramide synthesis, particularly SPT and specific isoforms of CERS (1, 5 and 6), leading to an increase in C16:0 ceramide (33–35). Conversely, adiponectin, by increasing ceramidase activity, elicits ceramide deacylation, thus promoting sphingosine formation and reducing the accumulation of these sphingolipids (**Figure 1**).

## 3 Ceramide accumulation at the nexus between adipose tissue dysfunction and systemic metabolic deregulation

The adipose tissue funnels excess energy availability towards the synthesis of triglycerides. While the majority of fatty acid excess is channeled towards the synthesis of triglycerides, a portion of lipids can be diverted towards the synthesis of sphingolipids, such as ceramides. In particular, ceramides, compared to other and more abundant sphingolipids, apart from being more tightly associated with poor cardiometabolic and health (16, 25) are also more susceptible to fluctuation in fatty acid bioavailability (36). Ceramide levels and types in the circulation and within metabolically active tissues, such as the liver and the skeletal muscle, closely reflect the metabolic status of an individual being associated with obesity and insulin resistance in humans (37–39). The adipose tissue is no exception, with an increase in ceramide buildup in this tissue being reported in individuals affected by obesity (13, 34). However, adipocytes are able to accumulate a considerable amount of triglycerides which makes them less susceptible to saturated fatty acid-induced ceramide accumulation relative to myotubes (40). Thus, as far as adipocytes retain the capacity to store excess saturated fatty acids, such as palmitic acid, in the form triglycerides, their insulin sensitivity is preserved (41). However, overwhelming adipocyte lipid storage may shift fatty acid excess towards ceramide synthesis fostering the accumulation of these sphingolipids. Thus, despite the adipose tissue being specialized in storing lipids, excess ceramide accumulation can still hamper its metabolic homeostasis contributing to systemic metabolic dysregulation. In agreement with this paradigm, the buildup of ceramides within the adipose tissue has been associated with insulin resistance, the metabolic syndrome and T2DM in humans as well as animal experimental models (13, 42–44). Furthermore, adipose tissue CERS6 expression has been reported to positively correlate with adiposity while it was negatively associated with insulin sensitivity assessed by euglycemic clamp in the clinical setting (34). Finally, in mice, adipocyte depletion of *Serine palmitoyltransferase, long chain base subunit 2 (SPTLC2)*, in order to inhibit *de novo* ceramide synthesis, or the upregulation of acid ceramidase, to foster ceramide degradation, both improved insulin sensitivity (43, 45). Moreover, despite nutrient overload being pivotal in fostering ceramide synthesis, its accumulation may be independent of obesity. In line with this, despite the same degree of obesity, women with higher levels of adipose tissue ceramides, and particularly C24:1-cereramide, displayed higher liver fat accumulation (13). Similarly, ceramide levels were reported to be higher in metabolically unhealthy compared to metabolically healthy obese individuals (46). These findings suggest that defective adipose tissue lipid metabolism may lead to ceramide accumulation which, in turn, may represents an additional factor at the nexus between adipose tissue dysfunction and impaired systemic metabolic health. However, it must be acknowledged that ceramide metabolism within the adipose tissue differ between males and females. Indeed, ceramide accumulation within with gonadal adipose tissue was reported to be sexually dimorphic, with higher levels of these sphingolipids being detected in high-fat-fed male relative to female mice (47). In further support of these preclinical findings, this sex dysmorphic effect also impacts upon the type of ceramides accumulating within the adipose tissue as indicated by a selective increase in C18:1-cereramide in obese male and C24:1-cereramide in obese females (48). Finally, and most importantly, sex differences have also been described for the association between adipose tissue ceramide and insulin resistance, with a positive correlation being reported obese females but not males (48). Furthermore, female subjects with impaired glucose tolerance displayed an adipose tissue depot specific ceramide signature, as indicated by higher levels of C16:0; C18:0 and C24:1-cereramides in the visceral relative to subcutaneous adipose tissue (49). Thus, sex hormones are involved in regulating adipose tissue lipid metabolism thereby affecting ceramide accumulation. Nevertheless, further studies are warranted to elucidate the impact of sex dysmorphic ceramide metabolism within the adipose tissue on systemic insulin resistance.

## 4 Adipose tissue ceramide accumulation as a driver of insulin resistance and metabolic inflammation

**Figure 2:**
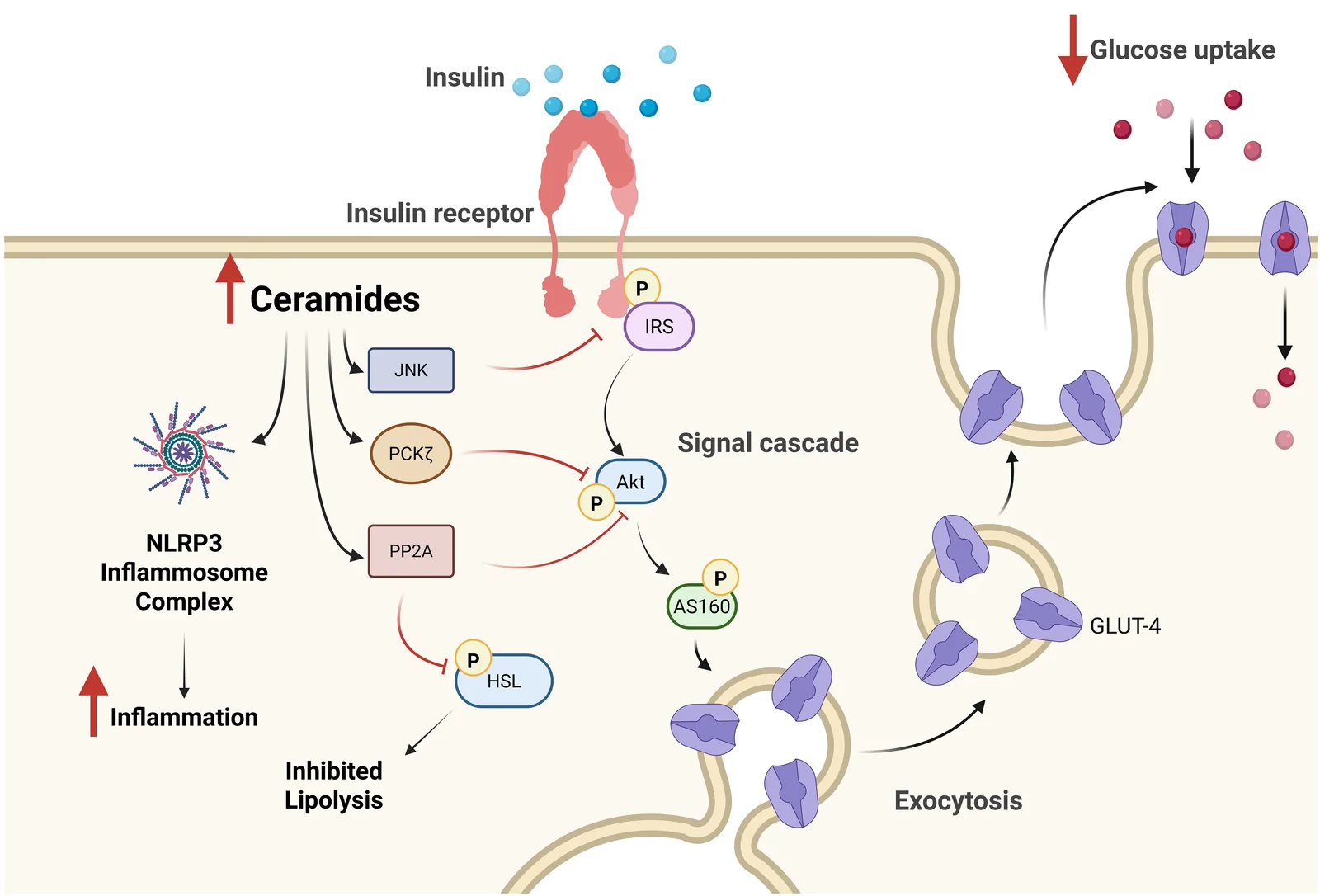
Molecular mechanisms underpinning the dysmetabolic effects of ceramide accumulation. Ceramideaccumulation within adipocytes promotes insulin resistance by hampering the phosphorylation andactivation of key proteins in the insulin signaling pathway. These effects are mediated by ceramide-induced activation of protein kinase C zeta (PKCζ), c-jun N-terminal kinase (JNK) and protein phosphatase 2A (PP2A). The two kinases (PKCζ and JNK) promote an inhibitory phosphorylation of protein kinase-B (PKB/Akt) and insulin receptor substrate (IRS), respectively, whereas PP2A promotes the dephosphorylation of PKB/Akt, thus hampering insulin signal transduction and glucose uptake. Moreover, PP2A inhibits β-adrenergic receptor-induced lipolysis by dephosphorylating hormone sensitive lipase (HSL). Finally, ceramide accumulation activates NOD-, LRR- and pyrin domain-containing protein 3 (NLRP3) thereby promoting inflammation and further fueling insulin resistance. AS160, Akt substrate 160 kDa; GLUT-4, glucose transporter 4. Image created with BioRender.com.

Ceramide accumulation within the adipose tissue recapitulates the pathophysiological mechanisms underpinning adipose tissue disfunction. Indeed, ceramides can hamper adipose tissue insulin sensitivity by disrupting insulin signal transduction pathway, mainly at the protein kinase B (PKB/Akt) level. The inhibition of this key node of insulin signaling is driven by ceramide-induced activation of atypical protein kinase C zeta (PKCζ) and protein phosphatase 2A (PP2A) (16, 25, 50) (**Figure 2**). While PKCζ elicit an inhibitory phosphorylation on Akt thereby preventing its activation and recruitment to the plasma membrane in L6 cells (51), PP2A promotes the dephosphorylation and inactivation of Akt in a variety of cell models (52). Nevertheless, due to the high abundance of caveolae in adipocytes, the PKCζ-dependent inhibition of Akt is preferred over the PP2A pathway, at least as far as cell models are concerned (15, 53). However, the inhibitory effects of ceramides on insulin signaling are not solely dependent on the inhibition of Akt. They also rely on the inhibition of insulin receptor substrate (IRS) mediated by the activation of c-jun N-terminal kinase (JNK) (**Figure 2**). This kinase phosphorylates IRS on serine 307 leading the inhibition of insulin signal transduction as demonstrated in murine and human myotubes (54). While ceramides can acutely dampen insulin signaling at the Akt level, a chronic exposure to these sphingolipids is required for their inhibitory effects at the IRS level (54). However, while plausible also in adipocytes, the inhibition of IRS in response to ceramide has only been reported in myotubes (54).

In addition to the impairment in glucose transported-4 (GLUT-4) -dependent glucose uptake, adipose tissue insulin resistance is also responsible for the inhibition of the anti-lipolytic effects of insulin. In line with this, ceramides, by hampering insulin sensitivity, may increase basal adipocyte lipolysis thereby increasing fatty acid availability to fuel lipotoxicity (55, 56). Thus, ceramides may be one of the mediators linking adipocyte hypertrophy and increased basal lipolysis. Nevertheless, while basal lipolysis is enhanced in individuals with obesity (55), catecholamine-stimulated lipolysis is reduced (57), with ceramides potentially countering contributing to this effect. These sphingolipids have been reported to inhibit β-adrenergic receptor-induced lipolysis by inhibiting hormone sensitive lipase (HSL) *via* PP2A in mice (58) (**Figure 2**).

Ceramides have a bidirectional relationship with inflammation, a key hallmark of adipose tissue dysfunction (59–62). Indeed, while ceramides can trigger inflammatory responses (63) and are associated with inflammation in human adipose tissue (64), the inflammatory mediator tumor-necrosis-factor-α (TNF-α) also promotes the synthesis of these sphingolipids as shown in MCF-7 cells (65). Data from animal models indicate that ceramides activate the NOD-, LRR- and pyrin domain-containing protein 3 (NLRP3) inflammasome in both adipocytes and macrophages (63) (**Figure 2**). Additionally, countering ceramide accumulation within mouse adipocytes has been reported to increase adipose tissue macrophages M2 polarization, suggesting these sphingolipids contributing to the crosstalk between adipocytes and immune cells infiltrating the adipose tissue (43). However, the role of ceramides in promoting the activation of the inflammasome in macrophages remains still controversial as another study reported that modulating ceramide biosynthesis selectively in these immune cells did not affect the inflammasome activation status (66). Furthermore, ablation of SPTLC2 in macrophages did not result in an improvement in glucose homeostasis (43, 58, 66), suggesting that the metabolically detrimental effects of ceramides are driven by their accumulation in adipocytes and do not rely upon macrophage endogenous synthesis. Thus, the dysmetabolic effect of adipose tissue ceramide accumulation appears to be mainly driven by adipocyte-derived ceramides and the secretion of these sphingolipids into the microenvironment, with EVs representing a potential vehicle for these lipotoxic lipid mediators. In turn, specific adipocyte-born ceramide species may target macrophages to modulate their polarization status. While this, may explain, at least in part, the controversial role of ceramides on the activation of inflammatory pathways within macrophages, the chemical characteristics of these sphingolipids may further dissect these discrepancies. Indeed, the controversial effects of ceramides on macrophage polarization appears to be partly dependent on their acyl chain length and saturation. In fact, long-chain ceramides have been reported to trigger the activation of the inflammasome (67) as well as the JNK and NF-κB pathways (68) and to prime cells to LPS-induced inflammation (69) in cell and animal models. Conversely, short-chain ceramides (C2:0 – C8:0) failed to trigger NF-κB activation unless elongated via metabolic pathways. These discrepancies may also be dependent on the use C2 or C6 ceramides, two short-chain sphingolipids analogues used in *in vitro* models, which may not fully recapitulate the biological properties of endogenous ceramides. Moreover, since the degree of lipid saturation strongly affects membrane fluidity and vesicles budding, the number of double bonds in ceramides may play a relevant role in macrophages polarization (70). However, the effect of ceramides on macrophages phenotype is mainly connected to the local microenvironment, which remains a major determinant of whether cells adopt M1, M2, or intermediate activation state. Despite this, macrophages infiltrating the adipose tissue may still indirectly contribute to the dysmetabolic effects of elicited by ceramides by secreting TNF-α which, in turn, may further promotes ceramide synthesis (65). The activation of the toll-like receptor (TLR)-4 has been proposed as an additional link between inflammation and ceramide synthesis. In agreement with this, the activation of the TLR4 in mice and RAW264.7 macrophages resulted in an increase in ceramide synthesis possibly dependent on the upregulation of ceramide-synthesizing enzymes (71, 72). Therefore, metabolic inflammation represents an additional mediator linking adipose tissue ceramide accumulation and poor metabolic health given the pivotal role of inflammation in the pathogenesis of insulin resistance (62, 73–76).

Ceramide synthesis, insulin resistance and metabolic inflammation are all interconnected (76) and should not be seen as independent entities. Indeed, enhanced ceramide synthesis must not be considered as a mere molecular trigger able to induce the activation of pathways which hamper insulin signal transduction and induce inflammatory responses. Instead, the upregulation of ceramide biosynthesis represents the cornerstone able to bridge the gap between overnutrition, and particularly long-chain saturated fatty acid overload, metabolic inflammation and insulin resistance (16). As such, while adipocyte being able to efficiently accommodate excess energy by storing it in the form on triglycerides, as metabolic substrate fluxes to the adipose tissue increase, a portion of energy surplus can be funnelled towards ceramide synthesis. Nonetheless, an increase in adipose tissue ceramide content in response to long-chain saturated fatty acid excess may also arise from enchanted ceramide trafficking from the circulation into adipocytes (50). Ceramide accumulation represents a direct link between the overconsumption of long-chain saturated fatty acids, and insulin resistance. However, while specific sphingolipids like long-chain ceramides (C16:0 and C18:0) being positively correlated with insulin resistance (77), this relationship may not necessarily be monodirectional. Indeed, defective adipose tissue insulin sensitivity may further fuel ceramide synthesis by increasing fatty acid bioavailability due to impaired lipolysis inhibition (15). However, it remains to elucidate whether increased fatty acid availability, due to unchecked lipolysis, fuels ceramide synthesis also in the adipose tissue beside the liver and skeletal muscle.

Furthermore, ceramides are pivotal in mediating the metabolic inflammatory response elicited by long-chain saturated fatty acids (15, 23). Thus, not only ceramides are able to directly hamper insulin signaling, but its metabolically detrimental effects on insulin sensitivity are also amplified by metabolic inflammation (15, 16). Most importantly, as already anticipated, not only ceramides are able to induce inflammatory responses, but inflammation itself further exacerbates ceramide synthesis (65). Thus, ceramide biosynthesis is emerging as a central player of a vicious cycle linking insulin resistance and inflammation with this intricated molecular network being further supported by the bidirectional relationship between ceramides and metabolic inflammation. Furthermore, saturated fatty acid-induced ceramide synthesis is required for the lipid-induced insulin resistance elicited by TLR4 activation (72), thereby further supporting this highly complex cross-talk between long-chain saturated fatty acid excess, ceramide biosynthesis, metabolic inflammation and insulin resistance.

## 5 Ceramide accumulation impacts upon adipose tissue secretome and extracellular vesicles secretion

As already anticipated, adipose tissue dysfunction is marked by a shift toward a pro-inflammatory, pro-insulin-resistant secretome, which is instrumental in linking obesity to its metabolic comorbidities (78, 79). This aberrant secretome is defined by the upregulation of pro-inflammatory cytokines (TNF-α, interleukin (IL)-6, -1β, -8) and adipokines associated with insulin resistance, including resistin (80–82). Concurrently, marked decrease in insulin-sensitizing adipokines, such as adiponectin (11, 83, 84) has been reported in both humans and animal models. In this context, the accumulation of ceramides within the adipose tissue, and particularly in adipocytes, may also contribute to shaping its secretome. In animal models, countering ceramide synthesis by *SPTLC2* ablation in rodent adipocytes led to an increase in the secretion of adiponectin, an insulin sensitizing adipokine (43). Similarly, to the findings in animal models, adipose tissue ceramide levels were negatively correlated with circulating adiponectin levels in females (48).

Besides modulating the secretion of classical adipokines, ceramides also impact on EV lipid cargo with these nanoparticles being a crucial component of adipose tissue secretome (8, 9). EVs represent small extracellular bodies originated from the blebbing of the plasma membrane or the endosomal pathway, sized between 30–200 nm and capable of carrying nucleic acids, proteins, to mediate inter-organ communication (12, 41, 85–87). However, it is also becoming evident that these nanoparticles also carry lipids (18, 88–90) as demonstrated in both rodents and humans. While according to the classical paradigm unesterified free fatty acids released via lipolysis represent the primary source of adipocyte-derived fat, EVs are now emerging as a lipase-independent shunt for lipid efflux from adipose cells (88). Consequently, lipids shuttled by EVs to target tissues may contribute to obesity-induced lipotoxicity and the onset of insulin resistance. Indeed, obesity not only increases the number of EVs released from human adipose tissue (91), but also reshapes their lipid profile, specifically by increasing ceramide content (18).

**Figure 3:**
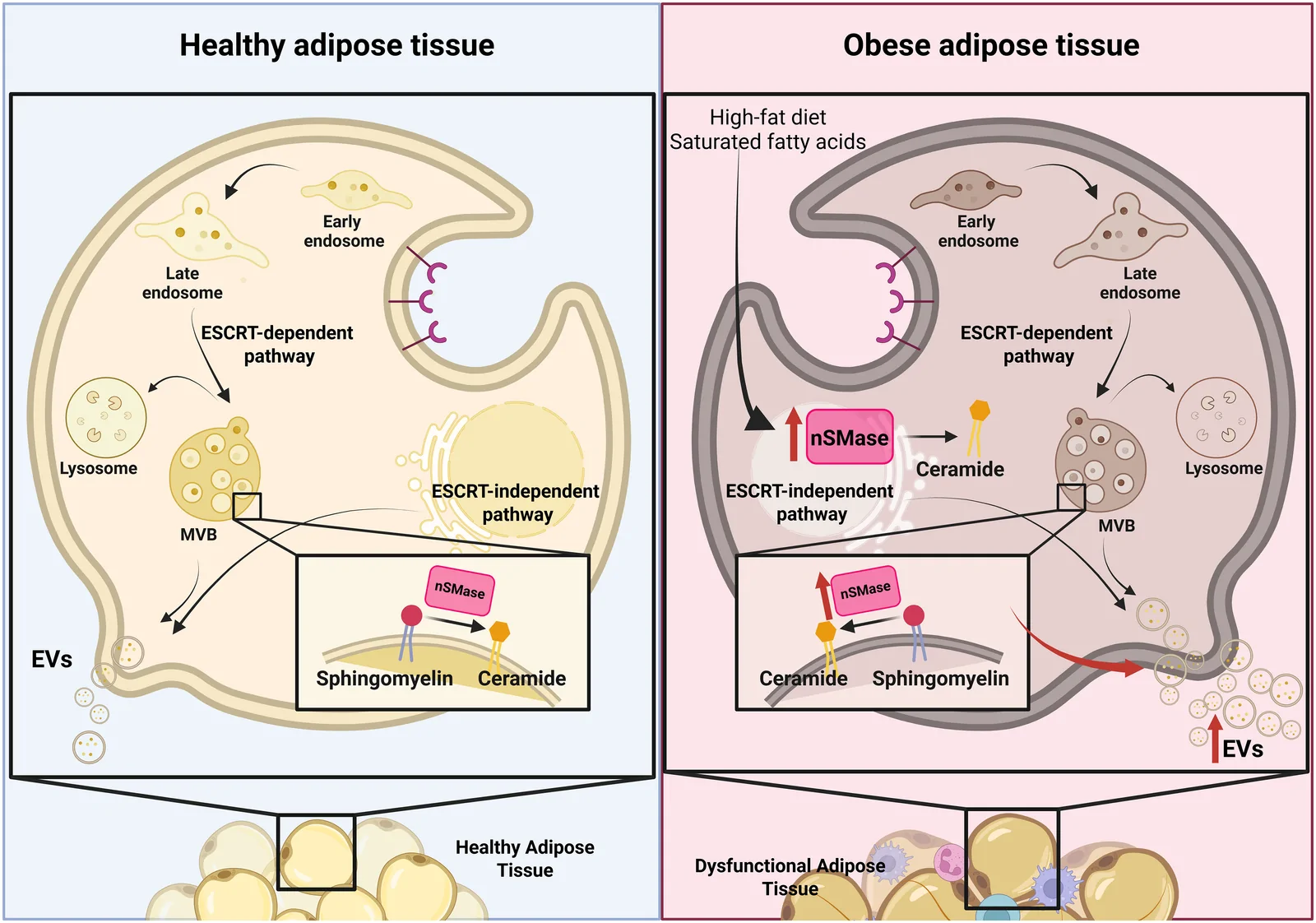
Ceramides are bioactive lipid components of extracellular vesicles. The biogenesis extracellularvesicles (EVs) relies on two different pathways, one dependent and the other independent ofendosomal sorting complex required for transport (ESCRT). ESCRT-dependent pathway requires endosome formation and maturation to multivesicular bodies (MVB) where membrane neutral sphingomyelinase (nSMase) transform sphingomyelins into ceramides, whereas the ESCRT-independent pathway relies on the activity of the nSMase associated to rugous endoplasmic reticulus (rER), responsible for ceramide synthesis through salvage pathway. While in healthy adipose tissue, there is a balance between the two pathways involved in EV biogenesis, the excess of saturated fatty acids responsible for adipose tissue dysfunction promotes an upregulation of nSMase resulting in an increase in ceramide production leading to enhanced EV biogenesis and secretion. Image created with BioRender.com.

Adipose tissue-derived EVs (AdEVs) possess a peculiar lipid signature that appears independent of vesicle size but linked to their membranous origin (85, 89). Frequently identified lipids within AdEVs include phosphatidylglycerols, ceramides and dihydroceramides, whereas phosphatidylserine, phosphatidylinositol, and phosphatidylethanolamine (PE) and its lyso derivates are typically less abundant (89, 90). Normally, EVs biogenesis requires Endosomal Sorting Complex Required for Transport (ESCRT)-dependent pathway (92). However, EVs can also be produced through and ESCRT-independent mechanism. This mechanism relies on the enzyme neutral sphingomyelinase (nSMase), with an increase in nSMase-mediated ceramide production leading to an increase in exosome release (17) (**Figure 3**). Notably, nSMase expression and activation is increased in response to a high-fat diet in rodents, thus an increase in ceramide accumulation in response to high-fat feeding is expected to increase EV secretion (**Figure 3**), as shown for liver-derived exosomes (93). Furthermore, increased hepatic EV secretion has been associated with an induction of nSMase activity (94) in both mice and humans and the upregulation of serine palmitoyl-transferase in mice with steatohepatitis (95). The accumulation of dihydroceramides within the adipose tissue was also associated with an increase in EV secretion in animals affected by obesity (89). However, not all ceramide species appear to contribute to the secretion of EVs from the adipose tissue to a similar extent. Indeed, EVs released from the adipose tissue of obese animals were enriched in 18:0/18:0 and 18:2/18:1 ceramides (89). Therefore, modulation of sphingolipid metabolism may represent a key mechanism by which a high-fat diet impact upon EV release from the adipose and other metabolically active tissues (90). Thus, independently on whether the synthesis of ceramides is mediated by nSMase or other anabolic pathways, the buildup of these sphingolipids within the adipose tissue may be involved in the uprise of AdEVs secretion in individuals with obesity. Furthermore, the visceral adipose tissue has been reported to secrete more EVs compared to its subcutaneous counterpart in obese individuals (7). However, it remains to be elucidated whether increased AdEVs secretion is attributable to fluctuations in adipose tissue ceramide levels. Thus, more studies are needed to disentangle the role of ceramides in EVs biogenesis in the context of obesity.

The lipid composition of the AdEV secretome does not simply mirror that of the adipose tissue. Instead, lipids are actively sorted into EVs depending on the adipose tissue depot they are secreted from and the metabolic status (89). Therefore, lipids in EVs are not only key components of their structure, while constituting a bioactive cargo component of these particles. Furthermore, the lipid signature of AdEVs is highly dynamic and intrinsically linked to the donor’s diet and metabolic status (89). In keeping with this, the shift in lipid composition of the visceral adipose tissue between healthy and obese states, marked by the upregulation of specific ceramides (49), is paralleled by a modulation of the lipid cargo of AdEVs. For instance, individuals with obesity display higher levels of saturated ceramides within the AdEVs, along with increased levels of saturated fatty acids (C16:0 and C18:0), cholesterol esters and acylcarnitines compared to their lean counterparts (18). Saturated ceramides cluster within cells membranes into lipid raft and influence membrane dynamics and viscosity (96). This, in turn, affects the downstream cell signaling as well as EV biogenesis (97). In keeping with this, saturated sphingolipids (C16:0 and C18:0), compared to their unsaturated counterparts, have been shown to enhance PKCζ activation, inflammation and ROS accumulation (98). The increase in lipotoxic species carried AdEVs suggests these nanoparticles acting as vehicles for lipids which may contribute to systemic metabolic deterioration. However, while obesity may lead to an increase in ceramides within the EVs released from human adipose tissue, the opposite was reported in rodents, particularly when considering the small EV fraction (89). Interestingly, no decrease in ceramide levels was observed when considering the EV secreted from the adipocytes of obese mice (89). This further suggests the contribution of adipose tissue to EV lipidome differing between adipocytes and the stromal vascular fraction (89). Nevertheless, it remains to elucidate the differences in terms of ceramide content in the EVs secreted from human versus mouse adipose tissue.

Diet quality, and particularly its lipid composition, adds a further layer of complexity to AdEVs lipid composition. In this regard, data from our (41) and other research groups (7) reported that palmitate-loaded hypertrophic adipocytes release more particles compared to untreated cells, with this effect potentially being mediated by an increase in ceramide synthesis. Moreover, those EVs showed increased levels of saturated fatty acids, particularly palmitate itself and its elongation product, stearic acid (41). This suggests EV lipid cargo being an additional mediator through which adipocytes inform other cells of their metabolic status, potentially contributing to lipotoxicity (41). The increase in palmitate within EVs could also be paralleled by an increase in ceramide content (99). For instance, palmitate treatment of non-adipose cells induced ceramide-enriched EVs, which may represent a mechanism intended to mitigate intracellular palmitate-induced cytotoxicity (100). Nonetheless, evidence of the role of nutrients and dietary patterns in modulating AdEV lipid composition are still lacking, with evidence available to date focusing on the effect of research high-fat diets in animal models (89) and palmitate overload in adipocyte models (7, 41).

While the accumulation of ceramides within the adipose tissue alters its metabolic health, AdEV may serve as a long-distance vector of ceramide-mediated lipotoxicity. Despite this being a plausible hypothesis, direct evidence of the metabolic effect of AdEV lipid cargo are lacking. Nonetheless, data on the EVs derived from other tissues support the functional effects lipidome contained within these nanoparticles. Indeed, the inhibition shingosine 1 kinase in hepatocytes reduced the sphingosine-1-phosphate-EV cargo, leading to a decrease in macrophage migration and ameliorating non-alcoholic steatohepatitis (101). Nevertheless, when evaluating these interorgan effects, it is critical to distinguish between direct functional transfer and associative human evidence. *In vitro* and animal models studies using lipid-tracers may provide direct mechanistic proof that AdEV-derived ceramides are functionally taken up by target recipient cells to trigger Lipotoxicity. Conversely, the human evidence available to date relies on correlations between circulating AdEV ceramide signatures and parameters of metabolic failure. Further tracing and targeted lipidomics studies are needed to quantify the exact fraction of tissue ceramide accumulation directly attributable to AdEV delivery *in vivo*.

AdEV ceramide content may also be regarded as a predictive biomarker of cardiovascular and metabolic diseases. Specific plasma ceramides (C16:0, C18:0, C24:0, and C24:1) have already been described as predictive markers of major cardiovascular events and coronary artery disease (25, 102). Lipids of AdEVs have also been proposed as predictors of metabolic dysregulation as highlighted using machine learning approaches (18). In support of this, AdEV lipid profiles can be used to train artificial intelligence to develop tools capable of discerning between an obese and a healthy lean metabolic phenotype with extremely high accuracy (18). In particular, ceramides within AdEVs were among the most critical lipids used by random forest models to predict the individual metabolic phenotype, with at least an 89% of accuracy (18). This further supports the relevance of EV ceramide content as a novel potential biomarker of cardiometabolic diseases. However, further research is needed to understand whether the enrichment of ceramides in AdEVs precedes the accumulation of ceramides in plasma and other metabolically active tissues.

## 6 Methodological challenges and future directions

The available evidence published to date only described an association between AdEV ceramides and metabolic deterioration in humans (18). However, it is currently unknown if AdEV can target other tissues to release their ceramide content and trigger lipotoxicity. As already described, AdEVs carry a plethora of bioactive molecules, such as miRNA, enzymes and adipokines, that have been shown to affect the metabolic health of recipient tissues (11, 103). Disentangling the metabolic effects of ceramides from those elicited by other bioactive molecules carried by EVs represents one of the main challenges in this field. Another potential explanation to this gap in the field is ascribable to some methodological criticalities. Classical EV-isolation methods, i.e. ultracentrifugation, size exclusion chromatography, precipitation do not allow particle fractioning except based on the size. For examples, assessing the role of EV-associated ceramides isolated from blood samples may be biased by co-precipitation and contamination from lipoprotein fractions which also contain ceramides (25). Several studies bypassed these limitations by switching to *in vitro* models based on cultured adipose tissue explants or adipocytes (88). Furthermore, some studies used adiponectin as marker to target and isolate AdEVs (11, 104), however this method does not allow to pinpoint EVs derived from a specific adipose depots. With this regard, adipose AdEVs exhibit distinct lipid sorting fingerprints that differ by depot origin, even though they express similar surface markers (89). Consequently, a step forward in this field will require targeted strategies to isolate depot-specific AdEVs, potentially by using specific lipid biomarkers. An additional cruvial step forwards would be the development of functional models capable of selectively manipulating ceramide cargo within these vesicles. Disentangling the specific bioactivity of EV-ceramides from other vesicular components (such as proteins and microRNAs) remains essential to fully define the independent contribution of these sphingolipids to metabolic health.

## 7 Conclusions

Ceramide accumulation within the adipose tissue represents a direct consequence of energy oversupply with its buildup possibly reflecting impaired adipocyte lipid metabolism and expandability. However, considering ceramide accumulation characterizing metabolically unhealthy individuals independently of obesity, ceramide accumulation may reflect defective adipose tissue lipid metabolism and not be a mere consequence of energy surplus. Ceramides may be at the nexus between energy overload and adipose tissue dysfunction being able to promote insulin resistance, metabolic inflammation and possibly impact upon adipose tissue secretome. Furthermore, these sphingolipids are a critical player in modulating EV secretion while being an important component of the lipid cargo of these nanoparticles. Despite the increase of ceramides in the EVs secreted from the adipose tissue of individuals with obesity with compromised metabolism, their pathophysiological role still remains to be determined. Indeed, further studies are warranted to evaluate whether the increase of these sphingolipids within AdEVs is a mere mirror of adipose tissue metabolic compromission or if it contributes to adipose tissue-drives systemic metabolic deregulation. In the latter case the enrichment of these lipid species within EV may represent a lipase-independent route through adipose tissue-derived ceramides are shuttled to other metabolically active organs to promote lipotoxicity and hamper metabolic health. Furthermore, these sphingolipids may function as a molecular shipping address guiding vesicles to recipient tissues. However, despite fascinating, these hypotheses remain to be tested.
